# Predicting the virulence of MRSA from its genome sequence

**DOI:** 10.1101/gr.165415.113

**Published:** 2014-05

**Authors:** Maisem Laabei, Mario Recker, Justine K. Rudkin, Mona Aldeljawi, Zeynep Gulay, Tim J. Sloan, Paul Williams, Jennifer L. Endres, Kenneth W. Bayles, Paul D. Fey, Vijaya Kumar Yajjala, Todd Widhelm, Erica Hawkins, Katie Lewis, Sara Parfett, Lucy Scowen, Sharon J. Peacock, Matthew Holden, Daniel Wilson, Timothy D. Read, Jean van den Elsen, Nicholas K. Priest, Edward J. Feil, Laurence D. Hurst, Elisabet Josefsson, Ruth C. Massey

**Affiliations:** 1 Department of Biology and Biochemistry, University of Bath, Bath BA2 7AY, United Kingdom;; 2 College of Engineering, Mathematics & Physical Sciences, University of Exeter, Exeter EX4 4QF, United Kingdom;; 3 Department of Clinical Microbiology, School of Medicine, Dokuz Eylul University, 35210 Konak, Turkey;; 4 Centre for Biomolecular Sciences, University of Nottingham, Nottingham NG7 2RD, United Kingdom;; 5 Department of Pathology and Microbiology, University of Nebraska Medical Center, Omaha, Nebraska 68198-5900, USA;; 6 Department of Medicine, University of Cambridge, Addenbrooke’s Hospital, Cambridge CB2 0QQ, United Kingdom;; 7 The Wellcome Trust Sanger Institute, Cambridge CB10 1SA, United Kingdom;; 8 Nuffield Department of Medicine, University of Oxford, Oxford OX3 7BN, United Kingdom;; 9 Department of Human Genetics, Emory University, Atlanta, Georgia 30322, USA;; 10 Department of Rheumatology and Inflammation Research, University of Gothenburg, 405 30 Gothenburg, Sweden

## Abstract

Microbial virulence is a complex and often multifactorial phenotype, intricately linked to a pathogen’s evolutionary trajectory. Toxicity, the ability to destroy host cell membranes, and adhesion, the ability to adhere to human tissues, are the major virulence factors of many bacterial pathogens, including *Staphylococcus aureus.* Here, we assayed the toxicity and adhesiveness of 90 MRSA (methicillin resistant *S. aureus*) isolates and found that while there was remarkably little variation in adhesion, toxicity varied by over an order of magnitude between isolates, suggesting different evolutionary selection pressures acting on these two traits. We performed a genome-wide association study (GWAS) and identified a large number of loci, as well as a putative network of epistatically interacting loci, that significantly associated with toxicity. Despite this apparent complexity in toxicity regulation, a predictive model based on a set of significant single nucleotide polymorphisms (SNPs) and insertion and deletions events (indels) showed a high degree of accuracy in predicting an isolate’s toxicity solely from the genetic signature at these sites. Our results thus highlight the potential of using sequence data to determine clinically relevant parameters and have further implications for understanding the microbial virulence of this opportunistic pathogen.

A key factor affecting the severity and outcome of any infection is the virulence potential of the infecting organism. If the virulence phenotype could be determined directly from its genome sequence, next generation sequencing technology would provide for the first time an opportunity to make predictions of virulence at an early stage of infection. Since the first whole-genome sequence of a free-living organism, *Haemophilus influenzae*, was published (Fleischmann et al. 1995), sequencing technology has advanced to a stage where a bacterial genome can be sequenced in a matter of hours (Parkhill and Wren 2011; Didelot et al. 2012a; Eyre et al. 2012; Köser et al. 2012a). This has led to an explosion of genomic data that has allowed us to monitor outbreaks in hospitals (Köser et al. 2012b; Young et al. 2012; Harris et al. 2013; Sherry et al. 2013; Walker et al. 2013), track strains transitioning from carrier to invasive status (Young et al. 2012), and perform detailed epidemiological studies to understand aspects of pathogen biology (Castillo-Ramírez et al. 2011, 2012; Didelot et al. 2012b; McAdam et al. 2012; Holden et al. 2013). While some success has also been made in predicting phenotype from genotype, such as the antimicrobial resistance (Farhat et al. 2013; Holden et al. 2013), for more complex phenotypes, such as virulence, involving the contribution of several genes, this has not yet been possible. Furthermore, complex interactions between genes (epistasis) are not apparent from genome sequences alone, nor is the effect of epigenetics (Borrell and Gagneux 2011; Jelier et al. 2011; Beltrao et al. 2012; Bierne et al. 2012).

*Staphylococcus aureus* is a major human pathogen, the treatment of which has been complicated by the worldwide emergence of multiple lineages that have acquired resistance to methicillin (methicillin resistant *S. aureus*, MRSA) (Lowy 1998; Gordon and Lowy 2008; Otto 2010). Its virulence is conferred by the activity of many effector molecules which can be broadly grouped into being either toxins (Lowy 1998; Gordon and Lowy 2008; Otto 2010)—factors that cause specific tissue damage in the host, or adhesins—factors that facilitate adherence to and invasion of host tissues (Foster et al. 2014). The ability of toxins to lyse human cells causes local tissue damage, facilitating immune evasion, release of nutrients, dissemination within a host, and transmission to others (Lowy 1998; Gordon and Lowy 2008; Otto 2010). A complex network of regulatory proteins controls the expression of many individual toxins (Priest et al. 2012), such that various sites on the *S. aureus* chromosome contribute to the overall toxicity of an individual isolate. The ability of *S. aureus* cells to bind human glycoproteins, such as fibrinogen and fibronectin, is another critical determinant in disease outcome. It facilitates attachment to and damage of host tissues, host cell invasion, and systemic dissemination (Foster et al. 2014). Several genes encode fibronectin- and fibrinogen-binding proteins (e.g., *fnbA*, *fnbB*, *clfA*, *clfB*, *eap*, *isdA*, *emp*, *ebh*, etc.), whose expression is again controlled by a complex regulatory network (Priest et al. 2012). Similar to toxicity, many sites on the chromosome can therefore contribute to the overall adhesiveness of *S. aureus*, with many regulators common to both adhesion and toxicity (Priest et al. 2012).

The success of epidemic MRSA clones such as USA300 and sequence type (ST) 239 is attributed to a variation in their expression of either toxins or adhesins (Li et al. 2010; Otto 2010; Li et al. 2012). In response to the prevalence of the highly toxic USA300 clone, guidelines exist that recommend treating suspected infections with vancomycin and a second antibiotic such as clindamycin or linezolid to reduce toxin expression and the associated disease severity (http://www.hpa.org.uk/webc/HPAwebFile/HPAweb_C/1242630044068). It is therefore clear that the ability to predict whether an infecting isolate is either highly adhesive or highly toxic could allow clinicians to adapt treatment approaches and increase their index of suspicion for disease complications for infected individuals.

To address this, we adopted a genome-wide association study (GWAS) and a machine learning approach to determine the feasibility of predicting virulence from the genome sequences of 90 MRSA isolates. Our findings demonstrate that using whole-genome sequence data for large collections of isolates to identify genetic signature associated with a specific trait can be used to infer complex phenotypes from genotype.

## Results

### Toxicity varies more than adhesiveness between *S. aureus* isolates

We first assayed the ability of 90 independent ST239 isolates (listed in Supplemental Table 1) to bind fibrinogen and fibronectin in both exponential and stationary phases of growth, as this varies and is believed to reflect different stages of infection. As expected, adhesiveness for all isolates was higher in the exponential than in the stationary phase (Supplemental Fig. 1A–D). However, across the 90 isolates only two differed significantly from the others, being higher in both growth phases. The limited variability of this virulence phenotype suggests it may be under strong purifying selection and would provide limited information on which to base a prediction of disease severity.

**Figure 1.**
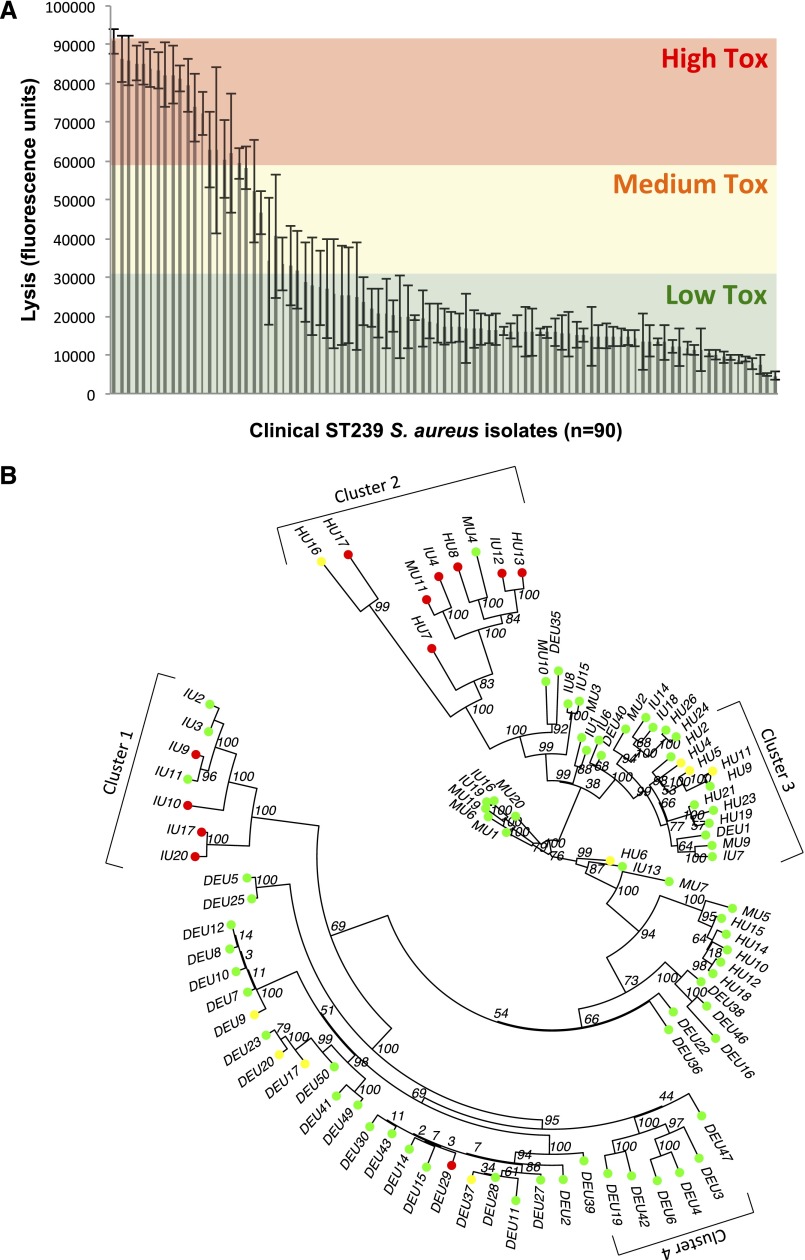
Toxic activity of clinical ST239 isolates. (*A*) The toxic activity of 90 ST239 isolates was assayed by incubating their supernatants with lipid vesicles containing a fluorescent dye. Dye release due to toxin-mediated vesicle lysis is determined using a fluorometer. (*B*) A maximum likelihood tree based on whole-genome sequences of the 90 isolates illustrating the distribution of the toxic activities of each isolate. Toxicity has been color-coded (red for highly lytic, yellow/amber for moderately lytic, and green for low level lysis). Clusters 1–4 are indicated for use in the stringent GWAS analysis.

We next measured the gross lytic activity of these isolates using an immortalized T-cell line (Collins et al. 2008; Rudkin et al. 2012) (sensitive to beta toxin, gamma toxin, delta toxin, LukED and PSMalpha1, alpha2 and alpha3) and lipid vesicles (Laabei et al. 2012) (sensitive to delta toxin and PSMalpha1, alpha2 and alpha3). No differences in lytic abilities were observed across these two assays (Supplemental Fig. 2), suggesting the effect is either largely PSM driven for the ST239 clone, or that the toxins assayed here are co-regulated. We also measured the expression of alpha toxin, as these lytic systems are not sensitive to this toxin’s activity, but no variation across the isolates was observed (Supplemental Fig. 3). However, the combined activity of the other toxins varied widely between the 90 isolates, with an 18-fold difference between the most and least toxic isolates (Fig. 1A). Interestingly, both the highly adhesive isolates identified above expressed low-level toxicity. (NB: This clone does not contain the Panton-Valentine leukocidin [PVL] containing phage [Castillo-Ramírez et al. 2012].)

To understand how differences in toxicity are distributed across the genetic variability that exists within this collection of isolates, we divided the data into three classes, scoring isolates as expressing either high (red: levels of >63,000 units), medium (amber: levels of 30,000:63,000 units) or low (green: levels of <30,000 units) toxicity. These three data ranges were selected so that a mid-toxicity range was included to account for possible cumulative effects of genetic polymorphisms. This was mapped onto a maximum likelihood tree based on the genome sequences of these isolates, showing a broad distribution of toxicity phenotypes across the genotypes as well as some clustering (Fig. 1B).

### Toxicity correlates with disease severity in vivo

**Figure 2.**
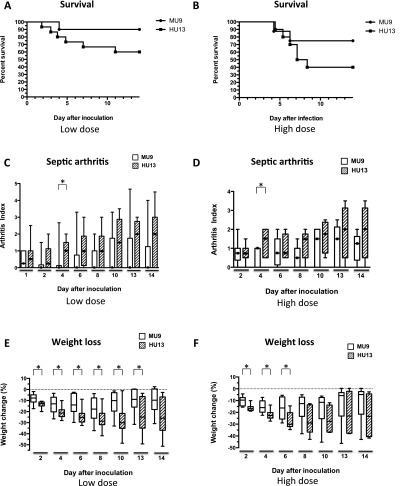
Predicted toxicity correlates with disease severity in vivo. Using high and low doses (7.8–8.0 and 3.7–4.1 × 10^7^ CFU, respectively), mice were inoculated intravenously with the high and low toxic isolates (HU13 and MU9, respectively), and survival of the mice, the development of septic arthritis, and weight loss were recorded as indications of disease severity. In each case the highly toxic HU13 isolate caused the most severe disease symptoms. (*A*) *n* = 10–15. (*B*) *n* = 8–10. (*C*) *n* = 10–20. (*D*) *n* = 10. (*E*) *n* = 10–19. (*F*) *n* = 10. Significant *P*-values (<0.05) are indicated (*).

To verify that toxicity correlated with disease severity, two isolates shown to have the highest and the lowest levels of toxicity in vitro (HU13 and MU9, respectively) were selected and their in vivo pathogenicity compared in a model of invasive infection (Josefsson et al. 2008; Kenny et al. 2009). Mice were injected intravenously with two different inoculum sizes; and murine survival, the development of septic arthritis, and weight loss were monitored over two weeks as a measure of disease severity (Josefsson et al. 2008; Kenny et al. 2009). Uninfected control mice did not die, did not develop septic arthritis, and did not lose weight over the duration of this experiment. In each aspect of disease measured here, the highly toxic HU13 isolate caused the most severe disease symptoms (Fig. 2A–F). It led to more deaths at both doses, although this was not statistically significant, caused significantly more severe arthritis at both doses at day 4, and resulted in significantly greater weight loss at both doses across many time points.

The isolates tested here are from the same sequence type but are not isogenic, and so other virulence-related traits may have played a role in the disease outcome. However, as toxicity is well established to affect disease severity, its variability even within this closely related group of isolates suggests that the ability to predict toxicity at an early stage of infection would be valuable clinical information.

### Identifying virulence-associated loci

We first employed a GWAS on the genomes of these 90 *S. aureus* isolates to identify the genetic polymorphisms that associated with the toxic phenotype. Out of a total of 3060 SNPs, we identified 100 that associated significantly with toxicity (with *P* < 0.05 after correction for genomic inflation [Supplemental Fig. 4; Supplemental Tables 2A, 2B], using a frequency cutoff for the occurrence of a polymorphism [i.e., successfully genotyped] across the population of >90% and a minor allele frequency of >5%). We further identified 22 toxicity-associated indels, using the same cutoffs for quality control. To test the effect of population structuring we used a hierarchical clustering algorithm (*pvclust*) in *R*, which showed strong bootstrap support for three main clusters (Supplemental Fig. 5), two of which contained all the highly toxic strains. We then performed a permutation procedure in PLINK, correcting for cluster membership, to obtain empirical *P*-values. Out of the 122 polymorphisms previously identified, only one (snp1360889) fell out using this procedure. Unfortunately, the limited sample size prevented us from using a more detailed clustering approach.

These SNPs and indels were distributed across the genome amongst mobile genetic elements, genes involved in metabolism and regulation, in hypothetical genes, and in intergenic regions. Two genes previously shown to affect the expression of toxins contained significantly associated SNPs: *mecA* (Rudkin et al. 2012) and *agrC* (Ji et al. 1995; Novick and Geisinger 2008), which provided some proof of principle for the validity of our approach. Mobile genetic elements, such as the *S. aureus* pathogenicity Island I (*Sa*PI1) (Ruzin et al. 2001) and the beta-haemolytic converting phage (Bae et al. 2006), also contained several associated genetic changes, implying that variability in many diverse regions of the genome contributes to the toxicity of a given isolate. Some of the polymorphisms appeared to be in linkage disequilibrium (Supplemental Fig. 6A), which will increase the rate of false positive associations, but many were uniquely occurring (i.e., unique patterns of polymorphisms across isolates) (Supplemental Fig. 6B).

This GWAS approach requires no evidence of repeatability of a signal, just an excess association between a SNP and the phenotype in question, and as such is likely to produce false positives with linkage disequilibrium and phylogenetic structure affecting the outcome. We therefore performed a second, more stringent approach, similar to those described in other recent work (Farhat et al. 2013; Sheppard et al. 2013), which instead requires repeatable independent evolution of a marker to be associated with the phenotype (toxicity). Although this approach should have a lower false positive rate, it is likely to produce a higher false negative rate. We focused on four clusters of isolates (indicated on Fig. 1B): cluster 1 (isolates IU20–IU2), cluster 2 (isolates HU16–HU13), cluster 3 (isolates MU2–IU7), and cluster 4 (isolates DEU3–DEU19). Clusters 1 and 2 contained the majority of the highly toxic isolates in this study, whereas clusters 3 and 4 represent the closest related clusters of low toxicity isolates to clusters 1 and 2. Where toxicity-associated polymorphisms are found in both clusters 1 and 2 but are absent from clusters 3 and 4 suggests that they have arisen independently. As such, they are likely to be causative as they are independent of phylogeny. Of the 121 polymorphic sites that associated significantly with toxicity, only four were found in both high-toxicity clusters (1 and 2) but not in their sister, low-toxicity clusters (3 and 4). All four of these polymorphisms (SNPs 78396, 2128192 and indels 2111134 and 2147199, see Supplemental Tables 2, 3) reside on mobile genetic elements, suggesting they may have been acquired horizontally. Of these four polymorphic loci, the *mecA* gene (in which SNP78396 resides) confers methicillin resistance and has previously been shown to affect toxin expression (Rudkin et al. 2012).

### Functional verification of effect of polymorphisms on toxicity

**Figure 3.**
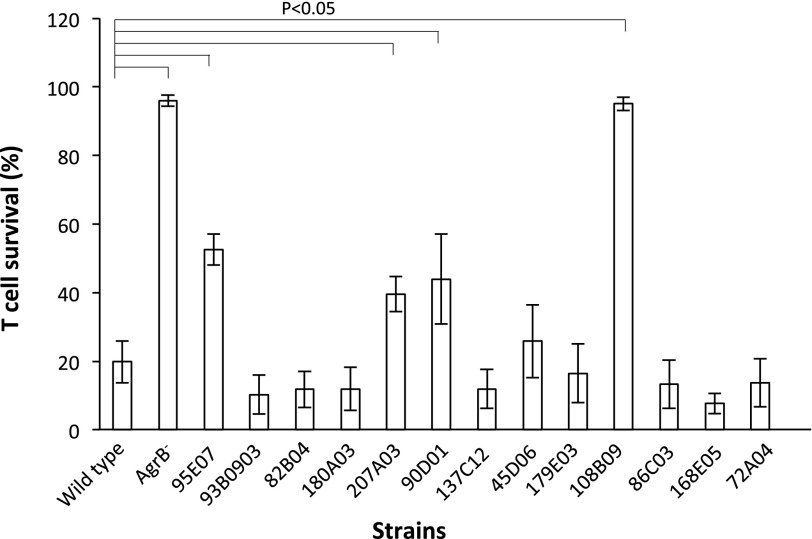
Functional verification using transposon mutagenesis. Mutated *S. aureus* isolates with transposon insertions in 15 of the 124 toxicity-associated loci were isolated (all in intergenic loci). Four of the 15 transposon insertions affected the toxicity of the isolate. The bars represent the mean % T-cell survival following incubation with bacterial supernatant, and the error bars the 95% confident intervals. Wild type represents the unmutated parent isolate, AgrB^−^ is a negative control, and the following are the transposon insertion mutants and their associated polymorphism: 95E07: 301089; 93B09: 761112; 82B04: 787629; 180A03: 799276; 207A03: 1121452; 90D01: 1503110; 137C12: 1931155; 45D06: 2027204; 179E03: 211134; 108B09: 2532617; 113D01: 2571739; 86C03: 2640325; 168E05: 2657438; 72A04: 2753734; 64A09: 2810368.

With the initial GWAS approach likely to produce a high number of false positive associations, we sought to obtain an estimate of this by determining the functional effect of a subset of these polymorphisms. We focused on 13 of the intergenic polymorphisms that could either affect the transcription of neighboring genes, or encode novel regulatory RNA molecules. We obtained transposon insertions in these polymorphic loci, ranging from 10 to 304 bp distal to the polymorphic site, and determined the effect of this insertion on the toxicity of the mutant. Four of the 13 insertions affected toxicity (Fig. 3) verifying that these loci contain toxicity-regulating activity. The SNP at position 301,089 (represented by the transposon insertion in strain 95E07 in Fig. 3) is in between the *tarK* and *tarF* genes that are involved in the synthesis of wall teichoic acids (Qian et al. 2006). The SNP at position 1,121,452 (represented by the transposon insertion in strain 207A03 in Fig. 3) is between a hypothetical gene and *fmt*, which is involved in methicillin resistance and autolysis (Komatsuzawa et al. 1997), both activities known to contribute to staphylococcal virulence. The SNP at position 1,503,110 (represented by the transposon insertion in strain 90D01 in Fig. 3) is in a locus annotated as a pseudogene in TW20, but as intergenic between genes encoding a TelA-like protein and a putative branched-chain amino acid transporter protein in FPR3757. The SNP at position 2,532,617 (represented by the transposon insertion in strain 108B09 in Fig. 3) is annotated in FPR3757 as intergenic between a hypothetical and an AcrB/AcrD/AcrF family protein-encoding gene; however, in TW20 it has been annotated as a hypothetical gene. Further molecular characterization is underway to determine the activity of these loci, but this work demonstrates that although this approach produces false positive associations, having looked at only 13 polymorphisms it has identified four novel toxicity-affecting loci.

**Figure 4.**
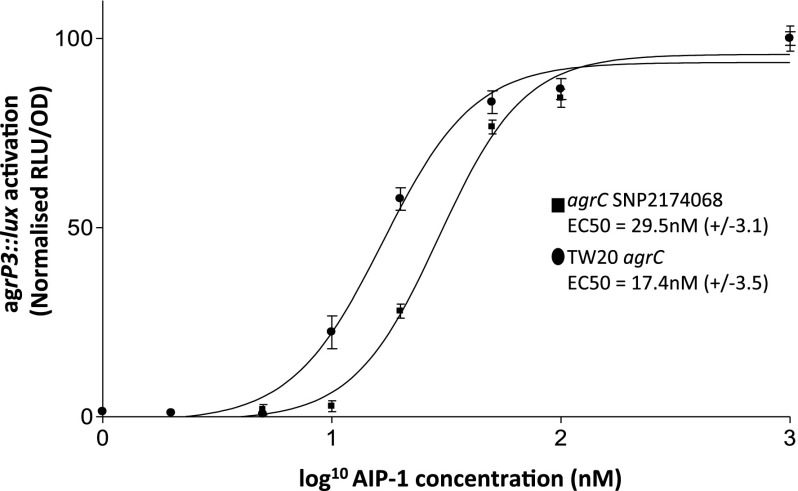
SNP2174068 has a major impact on the response of AgrC to AIP and hence toxicity. Dose-response curves for the activation of the *lux*-based *agr*P3 reporter via AIP-1 by the TW20 *agrC* allele (•) compared with the SNP2174068 variant (■).

As the more stringent approach described above yielded a shortlist of only four toxicity-affecting polymorphisms, we also sought to determine whether this approach, while reducing the false positive rate, would inadvertently dismiss potentially important loci. For example, a SNP in the *agrC* gene was identified by the initial approach as significantly associated with toxicity, but dismissed by the secondary more stringent approach. This protein forms part of a critical toxin regulatory system, and the SNP results in an A343T change to the amino acid sequence of the protein. The *agr* locus encodes a classical two-component regulatory system that allows the bacterium to regulate toxicity and adhesion through quorum sensing, in response to local cell density (Ji et al. 1995; Novick and Geisinger 2008). The AgrC protein is responsible for detecting the secreted autoinducing peptide (AIP) and transmits the signal to AgrA through phosphorylation. The phosphorylated form of AgrA acts as a transcriptional regulator at the *agr*P3 promoter of the Agr system, which drives the transcription of RNAIII, a regulatory RNA molecule, responsible for the regulatory changes that occur in response to the bacterial cells reaching a threshold density (Ji et al. 1995; Novick and Geisinger 2008). As such, this is a highly plausible candidate polymorphism that would have been disregarded by a more stringent approach.

The particular nucleotide change described here had not been identified previously, although other polymorphisms in the *agrC* gene have been shown to delay activation of the Agr system and as a consequence reduced the toxicity (Traber et al. 2008). Using a reporter system we evaluated the impact of SNP2174068 on the function of AgrC with respect to activation by exogenous AIP (Jensen et al. 2008). We compared the response of AgrC from the ST239 isolate TW20 with the AgrC encoded by the SNP2174068 containing *agrC* variant, by determining the half maximal effective concentration (EC_50_) of exogeneous synthetic AIP-1 for both (Fig. 4). The EC_50_ for the TW20 allele was 17.4 ± 3.5 nM, but almost twice as much AIP (29.5 ± 3.1 nM) was needed for the SNP2174068 containing AgrC variant, which suggests that, like previously identified polymorphisms in *agrC*, SNP2174068 delays the activation of the Agr system and as a consequence reduces toxicity. This work functionally verified the contribution of this particular polymorphism to the toxic phenotype, which would have been disregarded by the more stringent approach.

### Identifying epistatic interactions associated with toxicity

**Figure 5.**
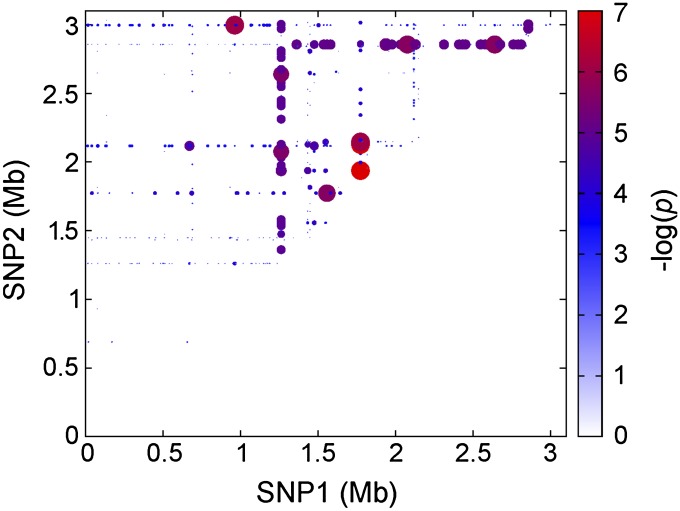
Heat-map representing interacting SNPs conveying epistasis between SNPs that affects an isolate’s toxicity. Each SNP is represented on both the *x-* and *y-*axes with the origin of replication based at the intersection of the axes (at zero). The size and color of the spot represent the significance of the interaction between SNPs as illustrated by the colored bar.

Genes and their protein products rarely act independently, with transcriptional, translational, post-translational regulators, and protein:protein interactions all playing a role in their activity. As a further hypothesis-generating exercise, we performed a pairwise test for toxicity-associated epistatic interactions on all combinations of SNPs and indels. A heat-map representing the genetic loci predicted to interact to affect toxicity is shown in Figure 5 (*P* < 1 × 10^−6^), where the size and color of each circle correspond to the statistical significance of the interaction, and in tabular form in Supplemental Table 3. Many of the interactions fell on straight lines, suggesting that a small number of genetic loci containing SNPs may be interacting with numerous other loci. From these we identified five genes that interacted with more than 20 other loci with high statistical significance: the *ileS* gene encoding isoleucyl-tRNA synthetase (Hurdle et al. 2004); the *mreC* gene involved in cell wall synthesis (Kyburz et al. 2010); an uncharacterized gene on the beta-haemolytic converting phage (Bae et al. 2006); the phytoene dehydrogenase gene, which is a key enzyme in the carotenoid biosynthetic pathway (Mijts et al. 2005); and a small, putative, regulatory RNA molecule (ssr100) (Anderson et al. 2006). Interestingly, the SNP in *ileS* has been shown previously to be responsible for conferring mupirocin resistance [V(588)F] (Hurdle et al. 2004), suggesting this may have pleiotropic effects on gene expression. The analysis also suggested that these loci also interact with one another, forming a novel and highly variable toxicity-regulating network.

However, caution must be exercised when interpreting these findings. As noted above, this approach is likely to produce a high number of false positives, and linkage between the SNPs that appear to be interacting with a single locus or population structure may affect the outcome of such analysis. For example, the SNP in *ileS* appears to be interacting epistatically with 30 other loci by this analysis. A more detailed survey of these 30 loci indicates that there are only nine independently occurring polymorphisms, which still suggests that *ileS* may have pleiotropic effects on the expression of other genes, but these need to be functionally verified before we can have full confidence in this interpretation.

### Predicting toxicity from genome sequence

**Figure 6.**
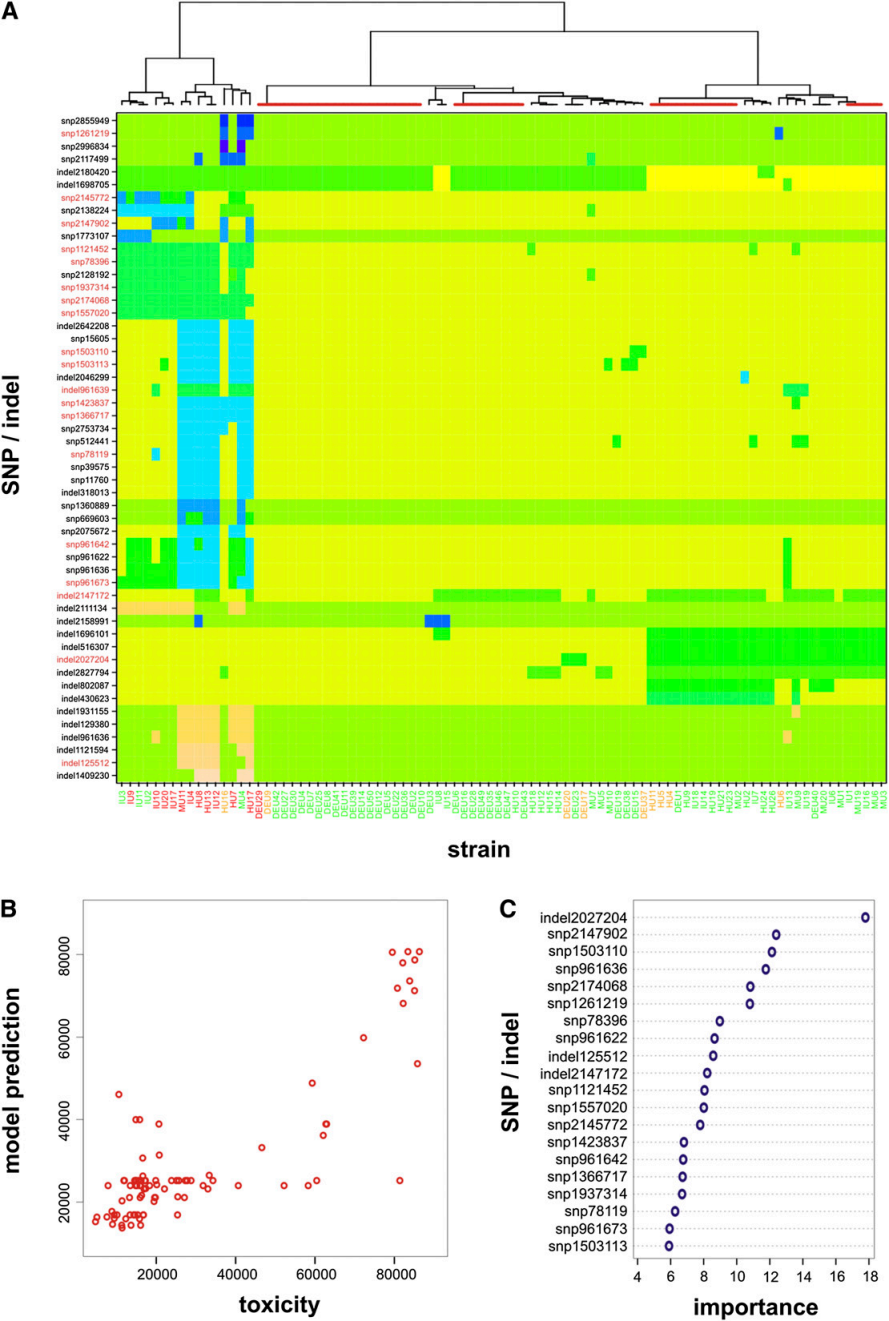
Genetic signatures affecting the toxicity of MRSA isolates. (*A*) Unsupervised hierarchical clustering analysis of significant SNPs/indels affecting toxicity in 90 isolates of the MRSA lineage ST239, color-coded (along the *bottom*) according to toxicity classes: low (green, <35,000), medium (orange, <65,000), and high (red, >65,000). Where an isolate has either the reference sequence at a site or the SNP/indel is illustrated as a change in block color across the rows. The most highly toxic strains are found to cluster together, indicating similar signatures independent of genetic background. Clusters highlighted by red bars on *top* denote strains with identical SNP/indel signatures. SNPs and indels highlighted in red (on the *left*-hand side) are those found to have high importance for the predictive model. (*B*) Random forest regression analysis shows a good fit between the strains’ observed level of toxicity and those predicted by the model; most outliers belong to clusters of identical strains, which cannot be resolved by these SNP/indel signatures. (*C*) Top 20 SNP and indels with highest influence on class prediction error, ordered by descending degree of importance.

Having identified specific genetic signatures (SNPs and indels) that associate with toxicity, we next investigated whether these signatures could be used to build a predictive model. Of the polymorphisms originally associated with toxicity, either directly or through epistasis, many were not unique but in complete linkage disequilibrium (Supplemental Fig. 4A). We therefore considered a subset consisting of all the unique SNPs/indels and one from each of the linked groups, which left 31 SNPs and 21 indels (Supplemental Fig. 4B). Performing a hierarchical cluster analysis on this subset highlighted two important aspects. First, all but one of the highly toxic strains (labeled red at the bottom of Fig. 6A) fall within the same cluster, indicating that these signatures are not simply based on the genetic relationship between the isolates (cf. Fig. 1B). Second, there are a number of strains with different levels of toxicity but with identical SNP/indel signatures; these form individual clusters (highlighted as red bars in the dendrogram on top of Fig. 6A) and can therefore not be resolved by a predictive model based on these signatures alone.

To build the predictive model, we utilized a “random forest” machine learning algorithm (Breiman 2001; Touw et al. 2013), which we used for both regression analysis and class prediction. This method, which creates an ensemble of decision trees and then uses the mean for predictions, produces unbiased error estimates without the need for cross-validation. For the class-predictive model, we used the categories described above: low (class 1, green), medium (class 2, amber), and high toxicity (class 3, red), respectively. Using this set of SNP/indels the model showed an accuracy of >85%, corresponding to an out-of-bag (OOB) error rate estimate of <15%. As shown in Table 1A, the majority of low and highly toxic strains were correctly identified by this model, whereas none of the medium toxic ones were predicted correctly. This was further highlighted when performing a regression analysis (Fig. 6B), where toxicity could be predicted with a high degree of accuracy for most of the low and highly toxic strains. The top 20 most important SNPs and indels determined by this approach (in terms of their influence on the model’s performance) are shown in Figure 6C, details of which can be found in Supplemental Tables 2 and 3 and are discussed later.

**Table 1.**
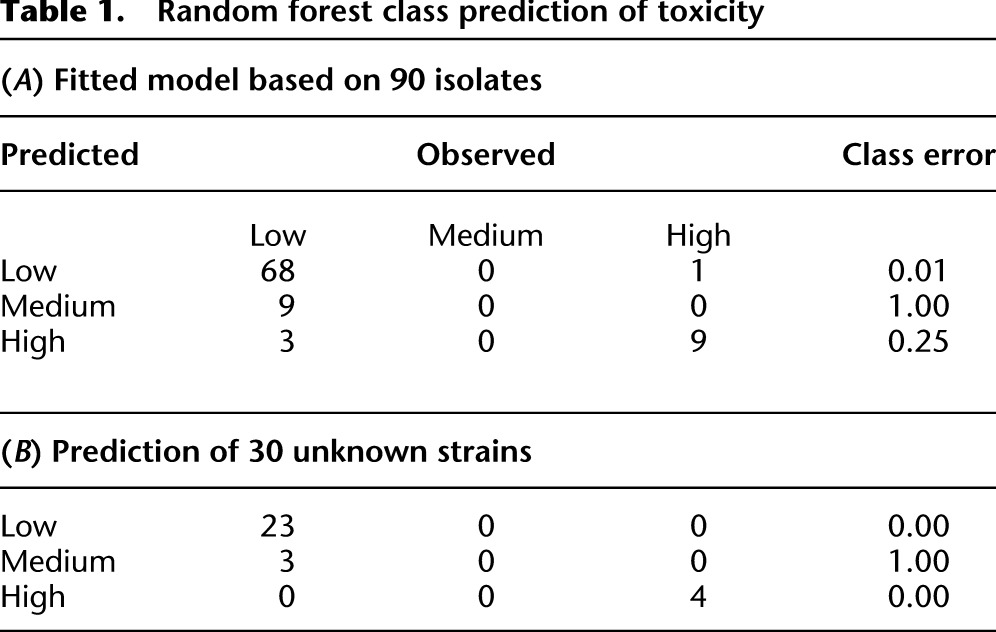
Random forest class prediction of toxicity

We further tested this method’s predictive ability by dividing the isolates randomly into a training set and a test set comprising 60 and 30 isolates, respectively. That is, we trained a random forest model on a subset of isolates, which we then used to predict the toxicity class of the remaining, and to the model unknown, test isolates. As shown in Table 1B, all of the low and highly toxic strains (23/23 and 4/4) were predicted correctly, whereas the strains of medium toxicity were exclusively underestimated. Although this clearly demonstrates the feasibility of our approach in predicting toxicity from genome sequence data, even in the face of unknowns such as epigenetic state, to be fully applicable to strains outside this clonal/ sequence type, the model would necessarily have to be trained on a much larger set of isolates from different genetic backgrounds.

## Discussion

The continuing emergence of drug resistant microbial pathogens is an issue of global importance. Although new drugs are being developed, their widespread use quickly selects for further resistance, which necessitates the development of approaches that allow clinicians to tailor treatment to a specific patient’s needs. Genomic data are believed to hold the key, but we do not yet have sufficient information to know which parts of the genome to examine to determine the best treatment strategy. While we are beginning to understand how to determine antibiotic resistance profiles from genome sequences, with hyper-virulent strains circulating we also need to understand how to determine the likelihood of an infecting strain to cause severe disease.

As toxicity and adhesion are key to disease outcome for *S. aureus*, we sought to determine their variability in a set of 90 isolates of the globally important ST239 clone, and whether these phenotypes can be predicted from genome sequences. Adhesion varied significantly in only two of the 90 isolates tested, and so for the majority of the isolates used in this study adhesion was entirely predictable without having to consider the genome sequence. Toxicity however, showed much greater variability between isolates, and given its importance in disease outcome became the main focus of this study.

GWAS has been widely used to identify genetic loci associated with human diseases. Although phylogenetic structure may affect the application of this to a prokaryotic system, GWAS is still a useful tool to identify candidate virulence affecting loci. Bacteria are haploid with high mutation rates, and so mutations affecting phenotypes are immediately detectable. Additionally, bacteria readily exchange DNA encoding virulence genes horizontally, and these are independent of phylogeny. With these considerations, we used GWAS and identified 121 genetic loci significantly associated with changes in the toxicity of individual isolates. Some of the genes in which this variability occurred have been identified previously as having a role in toxicity regulation, which demonstrated the validity of this approach. More importantly, it also identified a large number of novel putative toxicity affecting loci, and a set of five loci that appear to interact epistatically with each other and many other loci to affect toxicity, suggesting they may form a novel toxicity regulatory network. A more stringent approach reduced this list down to four candidate loci, and while this is a more manageable number to functionally verify, at least one functional locus (SNP in *agrC*) was lost by this approach. Although one method produced a high rate of false positives and the other dismissed potentially important loci, both have proven to be informative. When we attempted to functionally verify a subset (*n* = 13) from the long list of 121 by testing transposon insertion mutants in these regions, four proved to have toxicity regulating activity. This provided an indication of the false positive rate associated with the initial GWAS approach, and demonstrated that it is an effective means of prioritizing candidate genes for further functional characterization.

As an opportunistic pathogen, *S. aureus* can readily transfer from carriage to an invasive stage. It also has heterogeneity in its ability to transmit to new hosts, asymptomatically by direct contact or symptomatically through the production of pus. Adhesion is critical to all stages and transmission strategies. Toxicity, however, is more important for disease, pus production, and symptomatic transmission. Highly mutable loci in bacterial genes encoding proteins under strong immune selection are believed to have evolved to readily switch expression of the gene on and off, proving contingency for a fluctuating environment (Moxon et al. 2006). Here, however, we see great phenotypic diversity at the population level, encoded by many loci, the exact number of which can only be determined experimentally. With the relative benefit of toxicity being contingent on its life stage, it is therefore possible that having a complex regulatory system with many loci involved introduces the opportunity for great variability. This increased opportunity for variability in toxicity relative to adhesion may contribute to the opportunistic lifestyle of *S. aureus* in the same way a phenotype switch contributes at an individual level.

We adopted a machine learning approach and found that the presence of these loci was sufficient to predict the toxicity of the majority of isolates. This analysis also identified a list of highly important loci (Fig. 6C), the top of which was a 1-bp deletion in an intergenic region between the gene encoding the 16S ribosomal subunit and *perR*, a transcriptional regulator known to affect virulence (Horsburgh et al. 2001). Preliminary analysis of the sequence surrounding this site suggests it has a high level of secondary structure in a single stranded form, and the deletion of this base reduces this, which suggests it may be a regulatory RNA molecule, although further molecular analysis is needed to confirm this. The second most important site was a SNP in an uncharacterized gene on the *S. aureus* pathogenicity island 1, the third is in an intergenic region the effect of which on toxicity has been verified using transposon mutagenesis (Fig. 3), the fourth is in an uncharacterized gene on the beta toxin converting phage, and fifth is the *agrC* SNP characterized above. Although work to further characterize these loci and the role they play in toxicity is currently underway, this clearly indicates how this approach might be a useful tool for identifying new effector loci contributing to complex phenotypes.

The informative value of our hypothesis generating approach also extends to the case where there was a significant deviation between assayed toxicity and model prediction. That is, the toxicity of a small number of isolates was not well predicted, and we hypothesized that this could be explained by rare gain/loss-of-function genetic events that would not be identified using a statistical approach. A survey of all genetic changes associated with these poorly predicted isolates reveals that DEU29, for example, does not contain the beta-haemolytic converting phage (Bae et al. 2006). As such, unlike all the other isolates in this study, this isolate has an intact beta-haemolysin gene, providing a plausible explanation for why this is highly toxic despite being predicted as expressing low toxicity. MU4, which has a low level toxicity but was predicted to be highly toxic, has a unique SNP in the gene encoding the Rot (repressor of toxins) protein, which could have a dominant effect on toxicity (McNamara et al. 2000). The contribution of each SNP and indel event described here needs to be quantified in isogenic backgrounds, and although the scale of work involved is currently challenging, it is becoming more feasible with the development of high efficiency mutational protocols.

An alternative or complimentary explanation for the poor predictability for some isolates may lie in the mapping approach used. Illumina sequencing technology was used where the sequence data were mapped onto a reference genome, MRSA ST239 isolate TW20 (Holden et al. 2010). A limitation of this approach is that DNA not found in the reference strain is ignored, so additional genetic elements that could affect the toxicity of these poorly predicted isolates may not be identified. As sequencing on this scale improves with longer, better quality reads we will be able to perform de novo assemblies for each genome, which would allow all DNA in an isolate to be identified and tested for association with a specific trait.

It has been suggested that genome sequencing alone cannot give sufficient information to explain or predict complex phenotypes, as it does not consider the additional factors that affect protein expression such as epigenetics (Borrell and Gagneux 2011; Jelier et al. 2011; Beltrao et al. 2012; Bierne et al. 2012). However, here we have shown that using robust statistical techniques on large collections of sequenced isolates alongside machine learning approaches can yield desired results. When applied to virulence, while predicting the outcome of an infection will undoubtedly have to take into account the health and immune status of the affected host, we have described the first step toward this goal—that it is possible to predict the potential of a bacterial isolate to cause severe disease from the genome sequence alone. Further work quantifying the effect of each SNP on toxicity, virulence, and the expression of other virulence loci will add further detail to the model presented here. Also required will be the identification of more complex, three- and four-way epistatic interactions between genes, which will allow us to increase the model's predictive power. As the time nears when it is as cost-effective for a clinician to send a clinical sample for genome sequencing as it is to a routine diagnostic lab (Parkhill and Wren 2011; Didelot et al. 2012a; Eyre et al. 2012; Köser et al. 2012a), the next major challenge must be to adopt approaches as described here to build appropriate tools to convert genome sequences into information that can be used to help improve the treatment of infected patients.

We can imagine scenarios in which a patient’s bacteria are grown, targeted PCR, SNP arrays or rapid genome sequencing can be performed, and the machine learning approach applied to flag up, possibly within a few hours of initial bacterial isolation, whether the strain is likely to be toxic. The patient can then be immediately isolated, given virulence-modulating antibiotics, and monitored more stringently for complications. In addition to improving and personalizing the care of patients infected with highly toxic bacteria, it would also prevent the needless and deleterious administration of cocktails of potent and expensive antibiotics to patients with low toxicity infections. The predictive model itself would require regular updating, given all the new information. Whether there needs to be one model per clone, or one that adequately covers all isolates of *S. aureus* remains to be discovered. Either way, the approach described in this work is the first step in this direction.

## Methods

### Isolates and plasmids

The isolates and plasmids used in this study are listed in Supplemental Table 1.

### Fibronectin- and fibrinogen-binding assays

Bacterial adhesion to human fibronectin (Fn) and fibrinogen (Fb) (Sigma) was assessed using an adaptation of a previously published protocol (Edwards et al. 2010). For stationary phase growth, bacteria were grown for 18 h and were washed three times in phosphate-buffered saline (PBS). Final bacterial concentrations were normalized to an optical density of 0.5–0.55 at 600_nm_, which corresponds to ∼1 × 10^8^ CFU/mL. Exponential growth phase bacteria were grown for 3–4 h, with supernatant harvested and bacterial pellet washed and normalized as above. Adherent bacteria were calculated by using the crystal violet method (Edwards et al. 2010) and absorbance measured at A_595_ using a microtitre plate reader. Absorbance measurements were converted to bacterial numbers as described previously (Edwards et al. 2010).

### Toxicity assays

The toxicity of individual ST239 isolates was assayed in three ways. The expression of alpha toxin was determined by Western blotting using TCA precipitated 18-h bacterial supernatants (Ohlsen et al. 1997). No differences in signal intensity were observed across the 90 isolates (Supplemental Fig. 2). The ability of the isolates to lyse T cells, which measured beta toxin, gamma toxin, delta toxin, PSMalpha1, alpha2, and alpha3 activity was performed as described previously (Collins et al. 2008; Rudkin et al. 2012). Lipid vesicles, which are susceptible to delta toxin, PSMalpha1, alpha2, and alpha3, were prepared as described previously (Laabei et al. 2012). Briefly, vesicles for toxicity assay were composed of 25 mol% of 10,12-Tricosadiynoic acid (TCDA), 53 mol% 1,2-dipalmitoyl-sn-glycero-3-phosphocholine (DPPC), 2 mol% 1,2-dipalmitoyl-sn-glycero-3-phosphoethanolamine (DPPE), and 20 mol% of cholesterol (CHO). Lipid films were rehydrated in 50 mM 5(6)-carboxyfluorescein (CF) in HEPES buffer solution, freeze/thawed three times under liquid nitrogen extruded three times through 2 × 0.1 µm polycarbonate filters under nitrogen pressure. Vesicle purification was achieved through filtration through Nap-25 columns, stored overnight at 4°C and then cross-linked under UV for 6 sec. Toxicity assays were performed using 18-h bacterial supernatant and pure vesicles in a 1:1 ratio and fluorescence intensity measured at excitation and emission wavelengths of 485–520_nm_, respectively, on a FLUOstar fluorometer (BMG labtech). Positive and negative controls were pure vesicle with 0.01% Triton X-100 and HEPES buffer, respectively. No difference was observed in the lytic activity of the isolates whether vesicle or T cells were used (Supplemental Fig. 4), so the data from the vesicles are presented and were used for further analysis.

### Maximum likelihood tree

This was estimated using PhyML with an HKY85 substitution model, empirical nucleotide usage, no rate heterogeneity, and no invariant sites.

### GWAS

The identification of genetic variation in the clinical isolates studied has previously been described (Castillo-Ramírez et al. 2012). In summary, unique index-tagged libraries for each sample were created, and up to 12 separate libraries were sequenced in each of eight channels in Illumina Genome Analyser GAII cells with 75-base paired-end reads. Data have previously been deposited in the European Nucleotide Archive under study number ERP000228. The paired-end reads were mapped against the chromosome of *S. aureus* TW20 (accession number FN433596) (Holden et al. 2010) using SMALT (http://www.sanger.ac.uk/resources/software/smalt/) and SNPs and indels were identified as described in Croucher et al. (2011). For each isolate the average coverage ranged from 38- to 323-fold (stats for each isolate can be found in Supplemental Table 1), with a mean average coverage of 127 fold. Mobile genetic elements and accessory regions in the TW20 reference chromosome had previously been identified by manual curation (Holden et al. 2010).

We conducted a quantitative association study on a set of 90 isolates of the *S. aureus* clone ST239 to identify single nucleotide polymorphisms (SNPs) that were significantly associated with toxicity, using the PLINK software package (http://pngu.mgh.harvard.edu/purcell/plink/) (Purcell et al. 2007). From the original set of 3060 intragenic SNPs we identified 100 SNPs with statistical significance of *P* < 0.05 after quality control (using PLINK options -geno 0.9 and -maf 0.05) and correction for genomic inflation. A similar association study was performed using the indel data, where inserts, deletions, and wild types were coded as +1, −1, and 0, respectively. This identified 22 unique indels quantitatively associated with toxicity and present in at least five strains.

Analysis of SNP–SNP epistatic interactions was performed using the “epistasis” option in PLINK, which is based on linear regression analysis and tests the inclusion of an interaction term (into the regression equation) for statistical significance. Using a cutoff value of *P* < 1 × 10^−6^, we identified a further 20 SNPs that we included for the predictive model.

#### Transposon insertions

Transposon insertion clones of USA300 were obtained from the Nebraska Transposon Mutant Library (Fey et al. 2013).

### Class-predictive model

From the total set of 122 SNPs and indels we then removed those with identical “signatures” across the strains (see Supplemental Fig. 4), leaving 50 unique SNPs/indels which we used to build a class-predictive model. Due to the large number of free parameters and relatively low number of samples (i.e., isolates), we chose a random forest (Breiman 2001; Touw et al. 2013) approach, using the *randomForest* package in *R*, which is an ensemble machine learning algorithm based on decision trees. The benefit of this method is that it naturally provides generalization error estimates as well as variable importance, without the need for explicit cross-validation procedures (as these are intrinsic to the method). For class prediction we categorized our isolates based on measured toxicity into low (<40,000), medium (<63,000), and high. “Variable Importance” is automatically calculated by the algorithm by comparing, for each variable, the out-of-bag error rate for the final model fit to one where the variable is permuted. Larger differences therefore relate to higher importance.

### Site-directed mutagenesis of AgrC and construction of modified AIP/AgrC bioreporters

An *agr*P3∷*lux* bioreporter strain had previously been constructed by replacing the entire *agr* locus in RN4220 with the erythromycin resistance gene *ermB* and an *agrP3∷luxABCDE* promoter fusion to create ROJ48 (Jensen et al. 2008). A previously constructed plasmid pAgrP2C1A, containing the *agr*P2 promoter, *agrC*, and *agrA*, was then modified by site-directed mutagenesis to introduce either the I311T AgrC amino acid substitution found in the TW20 lineage, or both I311T and the A343T AgrC substitution conferred by SNP2174068. Mutagenesis was performed using the phosphorylated primers shown in Table 2 and Phusion DNA polymerase (New England Biolabs) before ligation of the resulting PCR products by Quick Ligase enzyme (New England Biolabs). ROJ48 was then transformed with the modified plasmids to create mutant bioreporters.

**Table 2.**
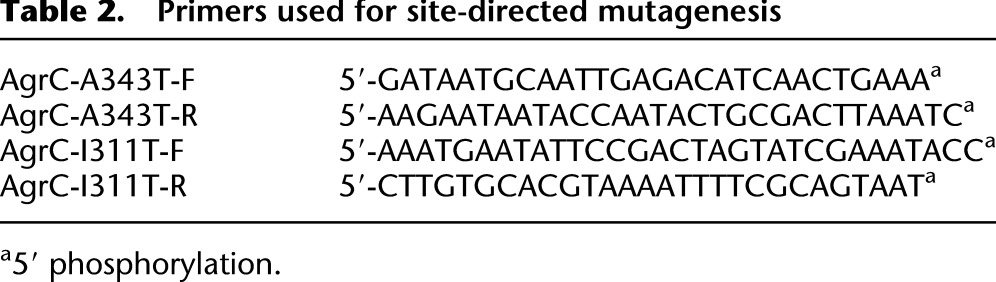
Primers used for site-directed mutagenesis

### AIP/AgrC bioluminescent reporter assay

The bioreporter strains TJS114 and TJS120, containing one of the mutated agrP2C1A plasmids, were grown overnight at 37°C in BHI medium supplemented with 10 µg/mL chloramphenicol. Overnight cultures were diluted 1:50 in fresh BHI before growth for a further 2 h and then diluted 1:20 into wells of a 96-well microtiter plate containing triplicate serial dilutions of AIP-1 in BHI. The plate was incubated in a Tecan microplate reader overnight and readings taken for relative light units and OD_600_ every 15 min. The two reporters with and without the A343T substitution were each tested in triplicate. Data were plotted as relative light units per cell density (RLU/OD) over time in Excel (Microsoft Corp.) and peak values from each concentration of AIP were extracted. Data for each reporter assay were normalized so that the RLU/OD at a saturating AIP-1 concentration (1 µM) was 100 and then exported to PRISM2 program (GraphPad). An EC_50_ value was then generated for each reporter based on the variable slope sigmoidal dose response curve.

### In vivo murine infection models

Female NMRI mice of 6–8 wk of age were obtained from Charles River Laboratories. Experiments were approved by the Animal Research Ethical Committee of the University of Gothenburg. *S. aureus* strains MU9 and HU13 were prepared for infection experiments as described previously (Josefsson et al. 2008; Kenny et al. 2009). Invasive infection was induced in mice by intravenous injection with a lower dose of strain MU9 (3.7 × 10^7^ CFU) or HU13 (4.1 × 10^7^ CFU), or with a higher dose of strain MU9 (8.0 × 10^7^ CFU) or HU13 (7.8 × 10^7^ CFU). Survival, arthritic index, and weight were monitored for 14 d. The overall condition of each mouse was examined by assessing signs of systemic inflammation such as weight decrease, reduced alertness, and ruffled coat. In cases of severe systemic infection, when a mouse was judged too ill to survive another 24 h, it was killed by cervical dislocation and considered dead due to sepsis. Clinical evaluation of septic arthritis was performed as described before (Josefsson et al. 2008; Kenny et al. 2009). Differences between groups were examined for statistical significance using the Logrank test at survival analysis, the Mann-Whitney test at arthritic index analysis, or the Student's *t*-test at weight decrease analysis. Arthritic index and weight change data are reported as medians, interquartile ranges, and 80% central range.
